# Neuromyelitis optica spectrum disorder secondary to treatment with anti-PD-1 antibody nivolumab: the first report

**DOI:** 10.1186/s12885-018-3997-2

**Published:** 2018-01-24

**Authors:** Yoshitsugu Narumi, Ryohei Yoshida, Yoshinori Minami, Yasushi Yamamoto, Shiori Takeguchi, Kohei Kano, Kae Takahashi, Tsukasa Saito, Jun Sawada, Hiroya Terui, Takayuki Katayama, Takaaki Sasaki, Yoshinobu Ohsaki

**Affiliations:** 1 Respiratory Center, Asahikawa Medical University, 2-1-1-1 Midorigaoka-Higashi, Asahikawa, Hokkaido 078-8510 Japan; 2 Department of Neurology, Asahikawa Medical University, Asahikawa, Japan; 3 Internal Medicine, Yoshida Hospital, Asahikawa, Japan

**Keywords:** Nivolumab, Neuromyelitis optica spectrum disorder (NMOSD), Lung cancer, Aquaporin-4, Immune-related adverse events (irAEs), Immune-checkpoint blockade, Programed death 1 receptor (PD-1)

## Abstract

**Background:**

Immune checkpoint blockade is developed as standard treatment for non-small cell lung cancer. However immune-related adverse events (irAE) have still unknown complications. Here, we report a patient with lung squamous cell carcinoma who developed neuromyelitis optica spectrum disorder with nivolumab.

**Case presentation:**

A 75-year-old Japanese man with lung squamous cell carcinoma was administered nivolumab as second-line treatment. Two months after treatment with nivolumab, he presented acute paralysis in the bilateral lower limbs, sensory loss. Spinal magnetic resonance imaging showed T2 hyperintense lesions between C5-6 and Th12-L1. He was diagnosed with neuromyelitis optica spectrum disorder (NMOSD) by anti-aquaporin-4 antibody-positive in the serum and other examinations. After treatment, steroid reactivity was poor.

**Conclusion:**

This is the first patient who developed anti-AQP4 antibody-positive NMOSD as a nivolumab-induced irAE. Clinicians should be aware of this kind of potential neurological complication by using immune check point inhibitor and start the treatment of this irAE as soon as possible.

## Background

Anti-programmed cell death protein 1 (PD-1) antibody nivolumab is now standard treatment as a second-line for non-small cell lung cancer [1, 2]. Although the frequency of adverse events is less than that of conventional chemotherapy, adverse events related to inflammatory response that have not been previously reported may occur. Here, we present a patient with lung squamous cell carcinoma who developed neuromyelitis optica spectrum disorder (NMOSD) with nivolumab.

## Case presentation

**Fig. 1:**
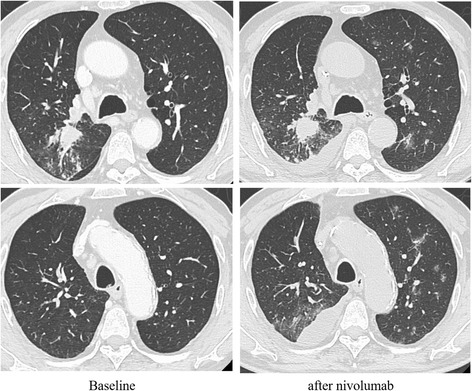
Clinical responses to nivolumab. Chest CT scan showed progressive disease after nivolumab

A 75-year-old Japanese man without any previous neurological illnesses was diagnosed with lung squamous cell carcinoma, stage IIIA, T3N1M0, in the upper right lobe. He underwent two cycles of platinum-based chemotherapy (carboplatin + nab-paclitaxel and carboplatin + docetaxel) as first-line chemotherapy. Thereafter, he was administered nivolumab (3 mg/kg) as second-line treatment. On the day of treatment with nivolumab, he had an infusion reaction as an adverse event. The cancer progressed after one cycle of nivolumab and chest computed tomography (CT) scan showed new lesions in the upper left lobe and an increase in right pleural effusion (Fig. 1) He underwent another round of platinum-based chemotherapy (cisplatin + pemetrexed + bevacizumab) as third-line treatment 3 weeks after the nivolumab treatment. However, he did not complete one cycle of the chemotherapy due to grade 3 constipation according to the Common Terminology Criteria for Adverse Events *version* 4.1.

**Fig. 2:**
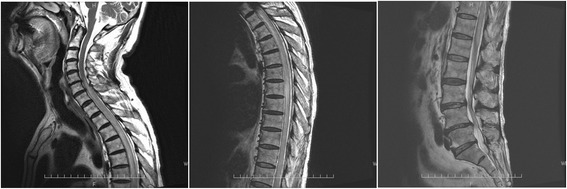
T2-weighted magnetic resonance imaging of the spinal cord showed longitudinally extensive intramedullary high-intensity areas (between C5/6 and Th12/L1)

Two months after treatment with nivolumab, he was hospitalized because of acute paralysis in the bilateral lower limbs, sensory loss below the Th10 level, and urinary retention. Spinal magnetic resonance imaging (MRI) showed T2 hyperintense lesions between C5-6 and Th12-L1 (Fig. 2). Analysis of the cerebrospinal fluid (CSF) showed an increased white cell count of 1195/μL (638 neutrophils and 557 mononuclear cells), protein concentration of 380.9 mg/dL, and a decreased glucose concentration of 40 mg/dL (blood glucose 139 mg/dL). CSF cytology was negative, and CSF cultures for bacteria, mycobacterium, and fungi were also negative. Polymerase chain reaction tests for herpesvirus 1–7 were negative. CSF tumor markers (carcinoembryonic antigen, squamous cell carcinoma, cytokeratin 19 fragment, and soluble interleukin-2 receptor) were all negative. Paraneoplastic autoantibodies (anti-Hu, Yo, Ri, amphiphysin, CV2, PNMA2, recoverin, SOX1, titin, zic4, GAD65 and Tr) were negative as well. An enzyme-linked immunosorbent assay (ELISA) and cell-based assay (CBA) for anti-aquaporin-4 (AQP4) antibody in the serum after hospitalization were positive, but these tests had been negative in the serum on the day of administration of nivolumab. CBA for anti-myelin-oligodendrocyte glycoprotein antibody in the serum was negative. Brain MRI and ophthalmologic examinations were normal. He was diagnosed with neuromyelitis optica spectrum disorder (NMOSD).

Two weeks later, after steroid pulse therapy, spinal MRI showed improvement in the rostral region, but a remnant lesion in the caudal region. Analysis of CSF showed improvement after treatment, but his symptoms of paralysis in the bilateral lower limbs and sensory loss improved only minimally. In this patient, steroid reactivity was poor. Plasmapheresis was performed, but his clinical symptoms did not improve. The patient’s paralysis in the bilateral lower limbs and sensory loss are gradually getting improved after plasmapheresis and continues rehabilitation for six months.

## Discussion and conclusions

Adverse events following immune checkpoint blockade are termed immune-related adverse events (irAEs), which are distinct from adverse events caused by conventional chemotherapy. Severe irAEs are rare, and grade 3/4 irAEs are present in less than 3% of patients [3]. Although reports of nervous system disorders such as autoimmune encephalitis and Guillain-Barré syndrome following immune checkpoint inhibitor therapy have been published [4, 5], no reports of nivolumab-induced NMOSD have been described. One report described a case of severe transverse myelitis in a patient with metastatic melanoma who was treated with ipilimumab, another immune checkpoint inhibitor, but anti-AQP4 antibody was not analyzed in this case [6]. A single case of demyelination associated bevacizumab has been reported [7]. However bevacizumab which is anti VEGF (vascular endothelial growth factor)-A agent ameliorates an animal model of multiple sclerosis [8]. Bevacizumab has therefore been proposed as a treatment strategy for NMOSD and Clinical trials of bevacizumab as treatment for NMOSD is still more in progress.

NMOSD is an inflammatory central nervous system syndrome that is associated with serum AQP4 immunoglobulin G antibodies (AQP4-IgG) [9]. CBA and ELISA (100% specific) yielded sensitivities of 68 and 60%, respectively, and sensitivity of 72% when used in combination [10]. In this case, ELISA and CBA for anti-AQP4 antibodies in the serum, which were negative immediately after treatment with nivolumab, became positive 2 months after treatment with nivolumab. This result strongly suggested that NMOSD was secondary to nivolumab. Although NMOSD is often recognized as optic neuritis, this patient only had paralysis in the bilateral lower limbs and sensory loss in the lower body as symptoms of acute myelitis. When early steroid pulse therapy is ineffective, the patient’s condition is often improved by plasmapheresis [11]. Although, spinal MRI and CSF indicated improvement, the patient’s clinical symptoms did not improve.

The precise mechanism causing NMOSD in this case remains unclear, but it is possible that nivolumab would have activated T-cells, especially interleukin-4-secreting Type 2 helper T-cells (Th2) and interleukin-17-secretring T-cells (Th17) leading to B-cell stimulation producing anti-AQP4 antibody and neutrophilic inflammation, based on recent surveys of the disease [12, 13].

To the best of our knowledge, this is the first patient who developed anti-AQP4 antibody-positive NMOSD as a nivolumab-induced irAE. Nivolumab-induced NMOSD may be refractory to treatment. Predicting emergence of NMOSD after nivolumab injection is currently difficult. Therefore, clinicians should be aware of this kind of potential neurological complication by using immune check point inhibitor and start the treatment of this irAE as soon as possible [14].

## Abbreviations

AQP4: Anti-aquaporin-4
CBA: Cell-based assay
CSF: Cerebrospinal fluid
CT: Computed tomography
ELISA: Enzyme-linked immunosorbent assay
IgG: Immunoglobulin G
irAE: Immune-related adverse events
MRI: Magnetic resonance imaging
NMOSD: Neuromyelitis optica spectrum disorder
PD-1: Programed death 1 receptor
Th17: Interleukin-17-secretring T-cells
Th2: Interleukin-4-secreting Type 2 helper T-cells
